# A randomised controlled trial of memory flexibility training (MemFlex) to enhance memory flexibility and reduce depressive symptomatology in individuals with major depressive disorder^☆^

**DOI:** 10.1016/j.brat.2018.08.008

**Published:** 2018-11

**Authors:** Caitlin Hitchcock, Siobhan Gormley, Catrin Rees, Evangeline Rodrigues, Julia Gillard, Inderpal Panesar, Isobel M. Wright, Emily Hammond, Peter Watson, Aliza Werner-Seidler, Tim Dalgleish

**Affiliations:** a Medical Research Council Cognition and Brain Sciences Unit, University of Cambridge, United Kingdom; b Cambridgeshire and Peterborough NHS Foundation Trust, United Kingdom; c The Black Dog Institute, Sydney, Australia

**Keywords:** Depression, Low-intensity treatment, Autobiographical memory, Memory flexibility, Randomised controlled trial

## Abstract

Successful navigation within the autobiographical memory store is integral to daily cognition. Impairment in the flexibility of memory retrieval can thereby have a detrimental impact on mental health. This randomised controlled phase II exploratory trial (*N* = 60) evaluated the potential of a novel intervention drawn from basic science – an autobiographical Memory Flexibility (MemFlex) training programme – which sought to ameliorate memory difficulties and improve symptoms of Major Depressive Disorder. MemFlex was compared to Psychoeducation (an evidence-based low-intensity intervention) to determine the likely range of effects on a primary cognitive target of memory flexibility at post-intervention, and co-primary clinical targets of self-reported depressive symptoms and diagnostic status at three-month follow-up. These effect sizes could subsequently be used to estimate sample size for a fully-powered trial. Results demonstrated small-moderate, though as expected statistically non-significant, effect sizes in favour of MemFlex for memory flexibility (*d* = 0.34, *p* = .20), and loss of diagnosis (OR = 0.65, *p* = .48), along with the secondary outcome of depression-free days (*d* = 0.36, *p* = .18). A smaller effect size was observed for between-group difference in self-reported depressive symptoms (*d* = 0.24, *p* = .35). Effect sizes in favour of MemFlex in this early-stage trial suggest that fully-powered evaluation of MemFlex may be warranted as an avenue to improving low-intensity treatment of depression.

**Trial registration:**

ClinicalTrials.gov, Identifier NCT02371291.

## Highlights

• Trial evaluates a mechanism-driven autobiographical memory-based intervention.
• Key aim was to prepare for a larger trial of MemFlex.
• Superior treatment effect sizes for MemFlex compared to Psychoeducation.
• A larger trial is now indicated to determine the treatment efficacy of MemFlex.

Clinical depression is a leading cause of disability (World Health Organisation, 2017), imposing a large economic burden estimated at approximately $210 billion a year in the US, with costs expected to increase in coming years (Greenberg, Fournier, Sisitsky, Pike, & Kessler, 2015). Our most efficacious psychological interventions for Major Depressive Disorder (MDD), Behavioural Activation and Cognitive Behavioural Therapy (CBT), are expensive to administer, requiring a therapist who has completed specialist training to work with an individual for a recommended 3–4 months for presentations of moderate severity (American Psychiatric Association, 2010; National Institute for Health and Care Excellence; [NICE], 2017). As such, waiting times for treatment can be long, with most individuals waiting an average of three months, and some not being able to access treatment at all (Docherty & Thornicroft, 2015). There is therefore a compelling need to improve access to effective psychological interventions for depression which can be delivered in a cost-effective manner with reduced therapist input, either as stand-alone treatments or as adjuncts to existing care programmes. Here we present a phase II randomised controlled trial (RCT) establishing proof-of-principle for one such putative low-intensity intervention – autobiographical Memory Flexibility Training (MemFlex).

Basic research into the psychological processes involved in the onset and maintenance of depression provides the ideal platform for the development of low-intensity clinical interventions. There is consistent empirical evidence that maladaptive styles of processing and remembering information are a driving force underlying depressive symptoms (Gotlib & Joormann, 2010), and effective psychological interventions, particularly CBT, seek to ameliorate depressogenic cognitive biases and thinking patterns and thereby improve symptoms. Autobiographical memory, that is, memory for personal life experiences, is one such system in which maladaptive cognitive biases predict and promote depressive symptoms (Williams et al., 2007). Theoretical models of autobiographical memory describe an information store that is arranged hierarchically, with generalised summaries of information available at the initial point of access, and event-specific information stored at the bottom of the hierarchy, such that autobiographical memories can be retrieved with varying levels of detail (Conway & Pleydell-Pearce, 2000). Depression is characterised by a tendency toward retrieval from higher levels of the hierarchy of ‘categorical memories’ which summarise consistent features across categories of past events (e.g., *when I visit my sister),* and a relative difficulty with voluntary retrieval from lower levels of the hierarchy of specific, single event memories which are highly contextualised in terms of time and place (e.g., *dinner at my sister's house last Sunday*).

Critically, for the point of view of putative intervention development, the degree of reduced accessibility of specific memories is a significant predictor of a poorer course of depression, over and above current levels of symptoms (Sumner, Griffith, & Mineka, 2010). This is theorised to be due to the importance of good specific memory access in problem solving, social interaction (Beike, Brandon, & Cole, 2016), and in modulating generalised pejorative self-judgements (Hitchcock, Rees, & Dalgleish, 2017a), and suggests that interventions designed to ameliorate these memory difficulties are likely to have therapeutic benefit. To that end, previous work (for review see Hitchcock, Werner-Seidler, Blackwell, & Dalgleish, 2017b) has demonstrated that structured intervention to improve memory specificity (e.g., Memory Specificity Training; Raes, Williams, & Hermans, 2009; Neshat-Doost et al., 2013) also improves depressive symptoms comparably to other evidence-based interventions (e.g., Werner-Seidler et al., 2018).

Other findings suggest that this difficulty in retrieving specific memories may be associated with a broader impairment in the intentional retrieval of either specific or generalised levels of autobiographical information. Dalgleish et al. (2007) demonstrated that depressive symptoms in a subclinical sample were also associated with poorer voluntary retrieval of categorical memories on a cued-recall task. Furthermore, in a sample of university students, Dritschel, Beltsos, and McClintock (2014) demonstrated that subclinical symptoms of depression were correlated not only with reduced retrieval of specific memories but also with difficulty alternating between retrieval of specific and generalised, categoric memories. We recently completed an experiment which demonstrated that relative to healthy controls, clinically depressed individuals experienced a large impairment (*d* = 0.90) in the ability to alternate between specific and categoric memory types. Notably, the impairment in memory flexibility was larger than that observed for memory specificity (*d* = 0.48) (see preprint; Hitchcock et al., 2018). Together, these findings suggest that it may not simply be that autobiographical retrieval lacks specificity but that those with depression may experience a more general difficulty in flexible navigation within the autobiographical memory store.

Successful navigation of autobiographical memory is likely to be important in supporting a number of cognitive processes that are central to daily life. The generalised summaries provided by categoric memories guide efficient decision making (Cosmides & Tooby, 2000; Klein, Cosmides, Tooby, & Chance, 2001), while specific memories play an important role in problem solving (Jing, Madore, & Schacter, 2016) and facilitating social interaction (Beike et al., 2016) – everyday skills which are compromised during depression, subsequently driving functional impairment. Improving the ease with which depressed individuals can generate these different memory types on demand, and move between them, may therefore help to alleviate symptoms of depression.

In addition to the demonstrated difficulties with targeted specific and categorical retrieval, depression is also associated with a chronic tendency toward recall of negative information and a relative neglect of positive information (Matt, Vazquez, & Campbell, 1992). Furthermore, when positive memories are retrieved, they appear to lack vividness and to fail to deliver the emotional benefits usually experienced upon recall of positive material (Werner-Seidler & Moulds, 2011). Impoverished quality of positive memories can thereby impair emotion regulation (Joormann & Siemer, 2004), while reduced accessibility provides fewer restraints on the scope of negative self-judgments that characterize depression (Hitchcock et al., 2017a). As a result, low-intensity interventions have also emerged to improve access to positive information, and there is some evidence of a beneficial effect on depressive symptoms (Williams et al., 2015). For these reasons, depressed individuals may benefit from intervention to improve the ease of access to and vividness of specific positive material, in addition to training flexible movement between positive and negative autobiographical memories of specific and categoric levels of detail.

Here, we introduce a low-intensity intervention – Memory Flexibility Training (MemFlex) – designed to improve targeted autobiographical memory retrieval in those with depression. MemFlex extends upon intervention programmes which have been successful in selectively targeting specific autobiographical memory recall (c.f., Memory Specificity Training; Raes et al., 2009) or reducing negative memory bias (c.f., positive imagery cognitive bias modification; e.g., Lang, Blackwell, Harmer, Davison, & Holmes, 2012) by simultaneously targeting both the valence and degree of specificity/generality of retrieved information. Memory Specificity Training has been shown to yield treatment effects that are comparable to, but not statistically superior to, those obtained using a counselling approach (Werner-Seidler et al., 2018). MemFlex aims to build upon, and hopefully improve treatment effects of existing autobiographical memory-based training protocols, by training memory retrieval more broadly. MemFlex targets intentional and flexible movement around the autobiographical memory store, thereby addressing both the specificity and valence of retrieval, with the rationale of mitigating the cumulative effects of multifaceted memory biases on depressive symptoms. Although the MemFlex programme is primarily self-guided (for meta-analysis of efficacy of self-guided programmes, see Cuijpers, Donker, van Straten, Li, & Andersson, 2010), it does include one guided-session that can be facilitated by individuals with minimal training in counselling or psychotherapy, followed by four weeks of self-guided workbook completion, in line with evidence that including a brief, non-individualised guidance component may make self-guided intervention more acceptable to service-users and yield greater treatment effect sizes than completely unguided self-help (e.g., Berger, Hämmerli, Gubser, Andersson, & Caspar, 2011). MemFlex is delivered via a hard-copy workbook and is brief (four weeks), and may thereby offer a cost-effective, accessible addition to the portfolio of psychological interventions for depression.

Translation of the MemFlex programme from basic science has followed recommendations for the phase-based development of novel interventions (MRC, 2000, 2008). An initial uncontrolled trial with individuals with recurrent depression demonstrated promising effects of MemFlex on memory retrieval (*d* = 0.48) and on processes through which autobiographical memory is proposed to impact depression (rumination, problem solving, and cognitive avoidance (*d*s from 0.18 to 0.55); Hitchcock et al., 2016). These preliminary findings, coupled with research indicating the beneficial impact of generating mental representations of vivid, positive episodes on core depressive symptoms such as anhedonia and a pervasive sense of worthlessness and failure (e.g., Blackwell et al., 2015; Hitchcock et al., 2017b) suggest that MemFlex may also help to alleviate depressive symptoms. The early-stage (phase II) RCT described here thereby sought to provide a controlled estimate of the effect of MemFlex on both cognitive and clinical outcomes in preparation for a later-stage, fully-powered trial.

We compared MemFlex to a guided self-help Psychoeducation Programme which included a number of factors that promote depression and are commonly addressed during CBT (e.g., sleep difficulties, procrastination, perfectionism), as psychoeducation is routinely the first option offered to individuals seeking psychological intervention for depression (Step 1 in stepped-care recommendations made by NICE, 2017). As the most likely role for MemFlex would be as a similar early-stage programme, either stand-alone or more likely as an adjunct or ‘primer’ to a more intensive psychological intervention (for instance, while patients are on the waiting list), a primarily self-guided Psychoeducation Programme that fulfils a similar role in stepped-care services seemed the relevant comparator.

The primary aim of this first RCT of MemFlex was therefore to estimate the likely effect of MemFlex relative to Psychoeducation on selected cognitive and clinical outcomes, in preparation for a potential later-phase, fully-powered definitive trial (MRC, 2000, 2008). We hypothesised effects in favour of MemFlex on a primary cognitive target of memory flexibility, indexed by improved accuracy on the Alternating Instructions version of the Autobiographical Memory Test (Dritschel et al., 2014) from pre-to post-intervention, and on co-primary clinical outcomes of diagnostic status (presence/absence of depression) and depressive symptoms (scores on the Beck Depression Inventory-II; Beck, Steer, & Brown, 1996), at three month follow-up. We also examined a secondary clinical outcome of number of depression-free days from post-intervention to three month follow-up (Hitchcock et al., 2015). Finally, we explored potential mediators and moderators of treatment effects.

## 1 Method

### 1.1 Protocol registration and publication

The trial was registered on clinicaltrials.gov (registration number NCT02371291) and the full trial protocol was published (Hitchcock et al., 2015).

### 1.2 Study design

**Fig. 1:**
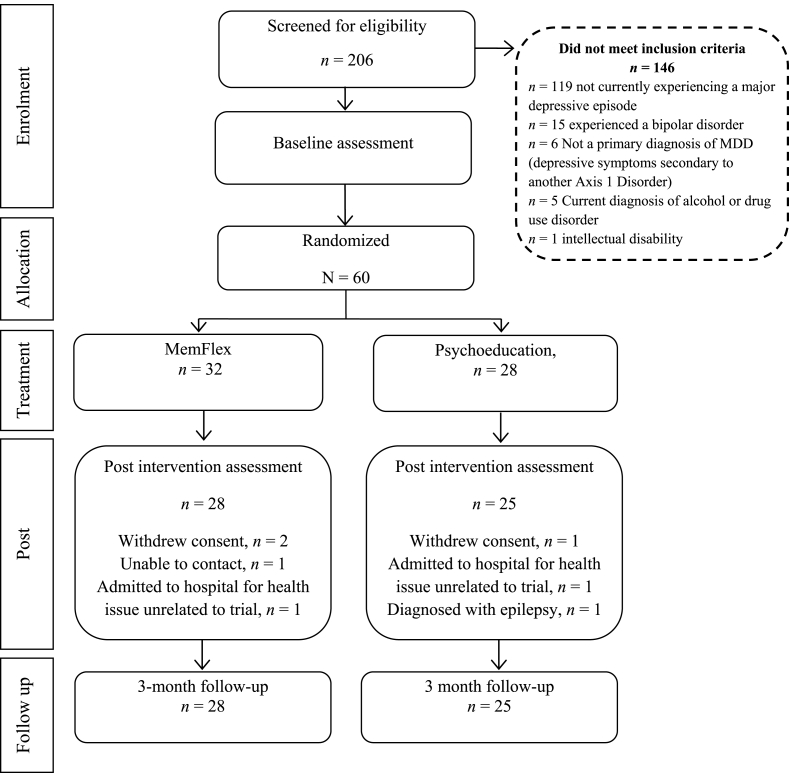
CONSORT diagram of study participation.

We completed a single-blind, patient-level, RCT comparing MemFlex to Psychoeducation. A CONSORT diagram of study participation is presented in Fig. 1. Each condition comprised an initial face-to-face session introducing the workbook which formed the main component of the intervention. Participants completed the workbook over the following four weeks. Assessments were completed at pre-intervention, post-intervention (the cognitive endpoint), and three-month follow-up (the primary clinical endpoint).

### 1.3 Participants and recruitment

For a definitive clinical trial, the standard approach to determine sample size is to complete a formal power calculation based on detecting an estimated treatment effect. The key aim of this early-phase trial was to provide an estimate of the size of the effect of MemFlex on plausible cognitive and clinical outcomes, such that this point estimate could be used to model the sample size for a later, fully-powered, definitive trial (MRC, 2000, 2008). Our prior experience with autobiographical memory-based interventions (e.g., Werner-Seidler et al., 2018) indicated that 24 participants per arm would provide sufficient numbers to estimate likely efficacy and acceptability, and provide a plausible range of point estimates for later work. We therefore recruited 60 participants to allow for 20% attrition.

Participants were randomised to MemFlex or Psychoeducation using a computer-generated random number allocation conducted by the trial statistician (Watson) who was blind to study objectives. Inclusion criteria were age over 18 years, diagnosis of Major Depressive Disorder, with a current episode, as determined by the Structured Clinical Interview for DSM Disorders (SCID; First, Williams, Karg, & Spitzer, 2015), and score ≥ 13 on the Beck Depressive Inventory- II (a minimum of ‘moderate’ symptom severity; Beck et al., 1996) at the eligibility screening. Exclusion criteria were current experience of psychosis, alcohol or drug use disorder (determined by the SCID), reported history of an intellectual disability or neurological disorder which may impact memory (e.g., epilepsy, dementia), and a lack of English fluency (assessed by the clinician). Participants were recruited in Cambridge, UK, through posters in community centres and health clinics, and through online and newspaper advertisements.^1^ Local NHS psychology services were also provided with information sheets about the study, and encouraged to hand these to suitable service-users. All interested parties emailed or telephoned the trial manager (Hitchcock) to express interest in participating. Following an initial phone screening, all eligible participants (see CONSORT diagram for number excluded) were invited to complete a SCID and the pre-intervention assessment at the research centre. All SCIDs were second-rated by the supervising clinical psychologist (Hitchcock), with 100% agreement on diagnoses.

### 1.4 Intervention

#### 1.4.1 MemFlex

The development of the MemFlex programme is discussed in detail in the trial protocol paper (Hitchcock et al., 2015). MemFlex is a primarily self-guided, paper workbook-based programme which teaches three key memory skills to improve the impairment in memory retrieval associated with depression. These skills are summarised as: *Balancing, Elaboration,* and *Flexibility*. Prior to completing one month of self-guided, workbook-based intervention, the participant attended one 45 min face-to-face session in which the facilitator outlined the importance of autobiographical memory in everyday life, discussed the impact of depression on autobiographical memory, and provided information on the different types of autobiographical memories (e.g., specific, categoric) and their potential everyday functions. The session also introduced the cued-recall tasks which were used to train the three key memory skills throughout the workbook, and provided facilitator-assisted practice with the tasks. Once participants were comfortable with the training exercises, they were required to set a schedule for completion of the eight-session workbook over the following four weeks. Halfway through this period, participants received a phone call from the facilitator to clarify any difficulties with the workbook material. Participants were also welcome to contact the facilitator with questions at any point. Fewer than 10% of participants contacted the facilitator outside of the progress phone call.

Session 1 of the workbook provided a review of the content of the face-to-face session. The *Balancing* skill was then introduced, which aimed to improve access to specific memories of positive and neutral emotional valence, to balance out the tendency for those with depression to retrieve memories that are negative and overgeneral. Participants were never explicitly asked to recall negative memories, but were required to provide memories in response to cues that may have prompted positive, negative, or emotionally benign autobiographical information (e.g., ‘travel’, ‘game’, ‘book’, ‘going for coffee with a friend’). *Elaboration* of the detail and emotion of recalled memories was introduced in Session 2. Session 3 consolidated both of these skills. Session 4 introduced *Flexibility* and guided the participant in moving between specific and general memories. Sessions 5 to 8 further consolidated the skills, aimed to improve the ease with which individuals could move flexibly between the memory types, and explored the application of the new skills to everyday life.

#### 1.4.2 Psychoeducation

Participants in the Psychoeducation condition also attended an initial face-to-face session, prior to completing a workbook consisting of eight self-guided sessions over a four week period (i.e., interventions were matched for time commitment). The initial session covered the symptoms and causes of depression, and introduced the workbook exercises. Participants also received a phone call from the facilitator at the beginning of week three to check progress, and clarify any difficulties with the workbook material. Again, participants were welcome to contact the facilitator with questions at any point, consistent with a guided self-help treatment format. Fewer than 10% of participants contacted the facilitator outside of the progress phone call.

The workbook sessions provided information on factors that perpetuate depression and are commonly addressed during CBT-sleep hygiene, worry, perfectionism, procrastination, anger, and assertive communication. The workbook content was closely based on the psychoeducational material provided by UK NHS psychology services, in an effort to create a rigorous comparison condition which was representative of current low-intensity treatment options. Session 1 provided a review of the material covered in the face-to-face session. Sessions 2–7 provided psychoeducation on one factor per session. Each session began with an overview of psychological theory underlying the target issue, and an explanation of the impact of the identified factor on depressive symptoms, followed by techniques to improve the factor (e.g., scheduling a ‘worry time’, setting a sleep routine). Each session included a series of multiple-choice questions about the material to ensure participant engagement, and an exercise requiring the individual to reflect on the role of that factor in their own lives, and on how they could use the suggested techniques. Session 8 required the individual to bring together all of the learnt material, and to make a plan to integrate their new knowledge into their self-care.

### 1.5 Measures

#### 1.5.1 Cognitive outcome

Our primary cognitive outcome was change in memory flexibility, indexed by change in the total number of memories correctly recalled in an Alternating Instructions version of the Autobiographical Memory Test (AMT-AI; Dritschel et al., 2014; Hitchcock et al., 2018), and in the number of correct memories in the alternating block. The AMT-AI is a cued-recall task which requires individuals to retrieve specific memories to a block of six cue words, categoric memories to a block of six cues words, and to alternate between retrieval of specific and categoric memories for a block of twelve cue words. Each cue was presented on a computer screen, along with task instructions, and participants were given 1 min to retrieve and verbally report the requested memory type. Responses were coded as of a specific event, categoric (i.e., an event that occurred on many occasions), extended (i.e., event lasting longer than one day), or repeated (i.e., a memory that had been previously reported), a semantic associate (i.e., information related to the cue which is not a memory), or an omission (i.e., could not think of a memory). Responses were scored as correct if the participant provided the requested memory type. Cue words were of positive, neutral, and negative valence, matched between-valence on frequency in the English language. Two wordlists were counter-balanced between pre- and post-intervention, stratified by intervention allocation, for each participant. Second scoring for 10% of AMT-AIs produced good (Cicchetti, 1994) inter-rater reliability – intraclass correlation coefficient = 0.78.

#### 1.5.2 Primary clinical outcomes

Our targeted co-primary clinical outcomes were depressive symptoms at three month follow-up, as reported on the Beck Depression Inventory II (BDI-II; Beck et al., 1996), and MDD diagnostic status at three month follow-up, measured using the Longitudinal Interval Follow-up Evaluation of the SCID (LIFE; Keller et al., 1987). The LIFE involves administration of the MDE criteria from the SCID, and indexes variation in depressive symptoms over the follow-up period using an ordinal symptom-based scale with categories defined to match the levels of symptoms.

#### 1.5.3 Secondary and additional clinical outcomes

The secondary clinical outcome was number of depression-free days from post-intervention to three month follow-up, also assessed using the LIFE.

Although they were not stated primary or secondary outcomes, because this was an exploratory trial where the aim was in part to estimate the range of clinical effects of our intervention, at pre- and post-intervention we also assessed symptoms of anxiety using the 21 item Beck Anxiety Inventory (BAI; Beck & Steer, 1993), a valid and reliable measure of state anxiety, and hopelessness using the Beck Hopelessness Scale (BHS; Beck, Weissman, Lester, & Trexler, 1974) which presents the individual with 20 true or false questions measuring negative attitudes towards the future.

#### 1.5.4 Additional process measures

As this was an exploratory trial we took the opportunity to administer counter-balanced measures at pre- and post-intervention of a number of factors through which autobiographical memory is thought to impact depression (see trial protocol, Hitchcock et al., 2015). The Means Ends Problem Solving Task (MEPS; Lyubomirsky & Nolen-Hoeksema, 1995) measures the ability to identify strategies for goal achievement. Participants were presented with an interpersonal problem scenario (e.g., not getting along with your partner) and asked to identify the ideal strategy for overcoming the problem. We utilised the shortened version of the MEPS (Scenarios 2, 4, 8 and 10) which is commonly used with depressed participants (Marx, Williams, & Claridge, 1992). Participants verbally reported their answers, which were audio recorded and later transcribed and scored. A score is given for the number of means used (i.e., steps taken) to solve the problem, along with a score out of seven for the likely effectiveness of the approach. Inter-rater reliability for 10% of responses was good (Cicchetti, 1994) for means (intraclass correlation coefficient = 0.76) and effectiveness (intraclass correlation coefficient = 0.76). The Cognitive Avoidance Questionnaire (CAQ; Sexton & Dugas, 2008) consists of 25 items which measure thought suppression, thought substitution, distraction, avoidance of threatening stimuli, and transformation of images into thoughts. Internal consistency for the total scale was good in the current sample, α = 0.80. The Ruminative Response Scale (RRS; Treynor, Gonzalez, & Nolen-Hoeksema, 2003) consists of 22 items that index the tendency to ruminate in relation to sad mood. We calculated both the total score and scores for the three subscales; self-focus, symptom-focus, and focus on possible causes and consequences of sad mood. Internal consistency for the total scale was good in the current sample, α = 0.83. Finally, the FAS Verbal Fluency Task (VFT) was administered as a measure of executive control in the domain of verbal information.

### 1.6 Procedure

Ethical approval was obtained from the NHS National Research Ethics Committee (East of England, 11/H0305/1). Written informed consent was obtained prior to completion of the SCID. Eligible participants then completed the pre-intervention assessment, which consisted of the AMT-AI, BDI-II, and process measures. We also administered the Digit Span Task (Wechsler, 2010) to allow us to assess whether general memory function at pre-intervention was comparable between conditions. The pre-intervention assessment and workbook session were completed on the same day. After completion of the pre-intervention assessment, the researcher opened a sealed opaque envelope that contained condition allocation and the workbook introduction session was completed, followed by the Credibility Expectancy Questionnaire – Patient Version (Devilly & Borkovec, 2000).

Treatment fidelity checks for 15% of audio recordings of the introduction session indicated 97.5% adherence to the respective manuals (for two participants, one in each condition, the facilitator did not ask the participant to complete a schedule for workbook completion). Post-intervention assessments were only conducted once the workbook had been completed, and occurred 4–6 weeks after the pre-intervention assessment (Overall *M* = 39.48 days, *SD* = 11.04, no between-condition difference *t*(52) = 0.65, *p* = .52). In addition to the LIFE, all primary and process measures were re-administered at post-intervention and wordlists for the AMT-AI, MEPS scenarios, and VFT categories were counterbalanced between pre- and post-assessments. Three months after the post-intervention assessment, participants completed the LIFE over the phone to index diagnostic status and depression-free days, along with a BDI-II which was emailed or posted back to the researcher. All assessors were blind to condition allocation, and participants were reimbursed £6 per hour for their time, in addition to a subsidy for travel fees. Clinical risk issues were managed by a clinical psychologist (Hitchcock). No adverse events were reported during the trial.

## 2 Results

### 2.1 Sample characteristics

Baseline means for participant demographics, primary cognitive and clinical outcomes, and process measures are presented in Table 1, Table 2. Correlations between these variables are presented in the Supplementary Materials. The only significant between-group difference was in the number of participants receiving a concurrent psychological treatment, with a higher number of participants receiving concurrent treatment in the Psychoeducation condition, χ^2^ (1) = 5.19, *p* = .03. In terms of previous depressive episodes, 19 individuals in the MemFlex condition and 17 individuals in the Psychoeducation condition had experienced too many prior episodes to distinguish the exact number, coded as ‘too many to count’ on the SCID. For those able to distinguish the exact number, the mean number of prior episodes was 3.82 (SD = 2.36) in the Psychoeducation condition and 4.08 (SD = 1.50) in the MemFlex condition. In terms of current comorbid diagnoses indexed by the SCID, 16 participants met criteria for generalised anxiety disorder, two met criteria for social anxiety disorder, one met criteria for specific phobia, six met criteria for posttraumatic stress disorder, one met criteria for bulimia nervosa, and two met criteria for obsessive compulsive disorder. At baseline, the mean percentage of correct responses on the AMT-AI was 57%, (95% CI [44.5, 69.5]) which was comparable to that obtained in prior depressed samples (62% correct, 95% CI [60.6, 63.4]; Hitchcock et al., 2018), and lower than that observed in never-depressed samples (83% correct, 95% CI [76.8, 88.6]; Hitchcock et al., 2018).

**Table 1:** *Mean (SD) participant characteristics at pre-intervention*.

	Psychoeducation (n = 28)	MemFlex (n = 32)^1^
Age	43 (17.60)	41.13 (16.21)
Number of females	19	22
Percentage Caucasian	60.71	68.75
Education history	2; 9;6; 8;3	1; 7;10; 10; 4
Percentage currently employed	42.86	40.63
Current Psychological treatment	14	7*
Current medication	19	24
Beck Depression Inventory-II	32.75 (11.63)	27.44 (10.09)
Beck Hopelessness Scale	13.46 (5.50)	11.69 (5.56)
Beck Anxiety Inventory	22.86 (11.76)	18.03 (10.87)
Digit Span	17.75 (4.18)	17.97 (4.06)

Note. *p = .03 for the between-group difference. For education history, highest level of education is 5th form; 6th form; undergraduate degree; postgraduate degree; diploma or professional training. For number of previous MDE, TMTC = too many to count, as coded during the diagnostic interview. 1 Human error in the randomisation procedure saw that numbers were not evenly allocated between conditions.

**Table 2:** Estimated Marginal Mean (SE) performance on the Alternating Instructions Autobiographical Memory Test and additional process outcomes at pre- and post-intervention, covarying for concurrent psychological treatment.

Variable	Psychoeducation		MemFlex		Indirect effect	
	Pre	Post	Pre	Post	*Β* (*SE*)	95% CI
AMT-AI Total	12.04 (4.58)	14.24 (5.59)	10.63 (5.59)	15.59 (4.23)	0.04 (.03)	[-0.002, 0.12]
Specific	3.64 (1.75)	3.76 (1.85)	3.25 (1.79)	4.30 (1.44)	−0.01 (.04)	[-0.12, 0.06]
Categoric	2.16 (1.70)	3.16 (1.31)	2.30 (1.54)	3.52 (1.85)	0.01 (.02)	[-0.03, 0.08]
Alternating	6.56 (2.92)	7.28 (3.53)	6.15 (2.92)	7.85 (2.01)	0.04 (.04)	[-0.01, 0.14]
Rumination	63.48 (9.89)	59.96 (11.24)	58.19 (9.32)	55.57 (10.50)	0.02 (.04)	[-0.05, 0.13]
Cognitive avoidance	76.70 (16.78)	76.70 (23.01)	69.72 (16.64)	67.76 (17.91)	−0.03 (.06)	[-0.16, 0.07]
Verbal Fluency	18.91 (4.60)	20.44 (4.90)	18.81 (5.80)	21.73 (4.72)	−0.01 (.03)	[-0.09, 0.05]
Problem solving means	3.46 (2.59)	6.33 (2.51)	3.74 (2.01)	4.44 (2.34)	−0.02 (.05)	[-0.12, 0.07]
Problem solving effectiveness	5.08 (2.36)	6.29 (1.81)	4.89 (1.97)	4.96 (1.93)	−0.03 (.06)	[-0.18, 0.06]

*Note.* Indirect effect is standardized. Specific and categoric are number correct out of six trials. Alternating is number correct out of 12 trials.

### 2.2 Intervention acceptability and feasibility

We achieved good adherence for a workbook-based intervention, with 84% of participants in the MemFlex arm and 89% in the Psychoeducation arm completing their workbooks – a completion rate comparable to or slightly higher than observed in trials evaluating computerised-CBT (Andrews, Cuijpers, Craske, McEvoy, & Titov, 2010). Non-completers were those did not attend the post assessment, and as such, we were unable to record how many workbook sessions they had completed. Participant ratings (from 1 = not at all, to 9 = very) on the Credibility Expectancy Questionnaire indicated that the workbooks were perceived to be broadly similar in how logical the offered treatment seemed (Psychoeducation *M* = 6.79, *SD* = 1.89; MemFlex *M* = 7.18, *SD* = 1.83), *t*(50) = 0.75, *p* = .46, *d* = 0.19 [-0.34, 0.72], and in how confident the individual would be in recommending the offered treatment to a friend (Psychoeducation *M* = 5.67, *SD* = 2.53; MemFlex *M* = 5.82, *SD* = 1.72), *t*(39.58) = 0.25, *p* = .80, *d* = 0.06 [-0.47, 0.59]. There was a moderate, though non-significant between-group effect in anticipated percentage symptom improvement, with MemFlex participants (*M* = 40.74, *SD* = 20.18) anticipating that they would experience approximately 10% greater improvement in symptoms than that anticipated by Psychoeducation participants, (*M* = 30.83, *SD* = 23.58); *t*(49) = 1.62, *p* = .11, *d* = 0.45 [-0.08, 0.98]. Similarly, there was a small-moderate effect in favour of MemFlex for how successful the offered treatment was anticipated to be (Psychoeducation *M* = 5.00, *SD* = 1.84; MemFlex *M* = 5.71, *SD* = 1.80), *t*(50) = 1.41, *p* = .17, *d* = 0.36 [-0.17, 0.89].

### 2.3 Analyses of primary and secondary outcomes

Analyses of the cognitive target, primary and secondary clinical outcomes were completed by the trial statistician (Watson) on an intent-to-treat basis using multiple imputation for missing data. We imputed 15 datasets for each missing value, in line with recommendations that the number of datasets approximates the percentage of missing data (Bodner, 2008). As noted, at pre-intervention, there was a significant difference between groups in the number of individuals receiving concurrent psychological treatment. As such, concurrent psychological treatment was covaried in all analyses. A Quade procedure (Quade, 1967) was also applied to correct for a violation in the assumption of normality within the completed ANCOVAs.^2^ This yielded a *t* value which was used to evaluate between-group differences on primary and secondary outcomes. Cohen's *d* and associated 95% confidence intervals were calculated from the *t* value obtained in the Quade procedure, and as such, provide the most accurate indication of the true effect. Conclusions regarding treatment effects should thereby be drawn from the Cohen's *d*, and estimated marginal means are presented for descriptive purposes only.

### 2.4 Cognitive target

We hypothesised that participants in the MemFlex condition would demonstrate greater improvement in AMT-AI performance from pre-to post-intervention, relative to Psychoeducation and here we sought to generate an effect size estimate for that difference. Descriptive statistics are presented in Table 2. We found a small to moderate-sized effect (Cohen, 1992) in the hypothesised direction for change in the number of correctly recalled memories during the AMT-AI, although this did not reach statistical significance, *t* = 1.29, *p* = .20, *d* = 0.34 [-0.19, 0.87]. A small effect size in favour of MemFlex was observed for improvement in the number of memories correctly recalled in the alternating block, *t* = 0.84, *p* = .40, *d* = 0.22 [-0.31, 0.75].

To further explore the impact of MemFlex on our cognitive target, we calculated within-group effect sizes for change from pre-to post-intervention. Significant, large improvements were observed in the MemFlex group from pre-to post-intervention in the total proportion correct, *d* = 1.03 [0.44, 1.62], *p* < .001, and in the proportion correct in the alternating block, *d* = 0.87 [0.29, 1.45], *p* = .003. A small-moderate effect size was observed for improvement in the Psychoeducation condition for the total proportion correct, *d* = 0.33 [-0.25, 0.91], *p* = .05, and alternating block, *d*=0.15 [-0.43, 0.72], *p* = .46.

### 2.5 Co-primary clinical outcomes

All participants experienced an improvement in depressive symptoms on the BDI-II from pre-intervention (*M* = 29.11, *SD* = 10.58, range = 10-56^3^) to follow-up (*M* = 22.66, *SD* = 10.69, range = 6–50), *F*(1, 45) = 19.44, *p* < .001, *d* = 0.91 [0.36, 1.46]. On average, participants decreased from the bottom of the Severe range to the bottom of the Moderate range (Beck et al., 1996). Across both arms, 55% of participants experienced minimally clinically significant improvement from pre-intervention to follow-up (minimum of 18% improvement in baseline BDI-II scores; Button et al., 2015). In terms of our co-primary endpoints, at follow-up, there was a small effect on remission rates (i.e., no longer meeting criteria for a Major Depressive Episode) in favour of MemFlex with 48% of the Psychoeducation condition having remitted, compared to 64% in the MemFlex condition, *OR* = 0.65, *d* = 0.24, although as anticipated for this exploratory trial this did not reach traditional statistical significance, *p* = .48. There was also a small effect size for a difference in BDI-II scores at follow-up between Psychoeducation (*M* = 23.86, *SD* = 11.09) and MemFlex (*M* = 21.60, *SD* = 10.44), in favour of the latter, *t* = 0.94, *d* = 0.24 [-0.29, 0.77], which again did not reach traditional statistical significance, *p* = .35.^4^ To index these treatment effects against recovery levels commonly observed in waitlist control groups (meta-analysis indicates an average pre-post assessment improvement of 15.7% in BDI scores; Posternak & Miller, 2001), we calculated within-group pre-to post-intervention effects for BDI-II scores. Both the MemFlex (24.3% improvement, *d* = 0.61, *p* = .007) and Psychoeducation (18.4% improvement, *d* = 0.46, *p* = .01) groups experienced a significant improvement in BDI-II scores from pre-to post-intervention.

### 2.6 Secondary clinical outcome

A small-moderate effect size in favour of MemFlex was observed for the proportion (arcsine transformed to stabilise variance) of depression-free days (i.e., number of depression-free days/number of days between assessments), *t* = 1.34, *p* = .18, *d* = 0.36 [-0.17, 0.89]. On average, those in the MemFlex condition achieved 14 extra days free from depression in the three months following workbook completion compared to those completing Psychoeducation, although again as expected this effect did not reach traditional statistical significance. On average, the MemFlex condition experienced 56% depression-free days in the three months following intervention, while the Psychoeducation experienced 43% depression free-days.

Exploratory analyses of additional clinical measures at post-intervention demonstrated small effect sizes in favour of MemFlex for the between-group difference in self-reported anxiety (*d* = 0.10) and hopelessness (*d* = 0.27), with neither effect reaching traditional statistical significance, *F*s < 1.

### 2.7 Additional process outcomes

We also explored the impact of intervention on the processes through which autobiographical memory impacts daily functioning; rumination, cognitive avoidance, fluent retrieval of verbal information (verbal fluency), and problem solving (see Table 2). Negligible, non significant effect sizes were observed for between-group change in rumination (*d* = 0.02), verbal fluency (*d* = 0.10), and cognitive avoidance (*d* = 0.11), *F*s < 0.14, *ps* > .71. A large and significant effect size in favour of Psychoeducation was observed for improvement in the number of steps used during problem solving, *F*(1, 48) = 8.96, *p* = .004, *d* = 0.84 [0.25, 1.43] (Bonferroni corrected *p* = .01), which flowed on to a moderate effect for increased effectiveness of problem solving, *F*(1, 48) = 3.99, *p* = .05, *d* = 0.56 [-0.01, 1.13], although this effect was not statistically significant following Bonferroni correction for the number of cognitive measures tested.

### 2.8 Exploratory posthoc analyses of treatment mediation and moderation

To explore the mechanisms underlying the effect of MemFlex on depression, we completed exploratory analysis of indirect effects on BDI-II at post-intervention through change in performance on the AMT-AI using 10,000 bootstrapped samples in PROCESS (based on observed data only; Hayes, 2012). Very small effect sizes (see Table 2) were observed for indirect effects, and confidence intervals for indirect effects spanned zero. Additional analyses for the other process measures revealed very small effect sizes for all indirect effects, and all 95% confidence intervals for indirect effects spanned zero (see Table 2).

Finally, we were interested in whether the impact of the different interventions would vary as a function of baseline symptom severity. Prior literature has indicated that baseline symptom severity can moderate the effects of process-based therapy (Kuyken et al., 2016), with the assumption being that those with more severe symptoms may demonstrate more strongly consolidated biases in processing. Clinically, such moderation effects would indicate whether MemFlex was more likely to be suitable for those with mild/moderate vs. more severe symptom severity. We therefore computed a product variable for group allocation × BDI-II score at baseline, and completed a hierarchical linear regression model predicting pre-to-post intervention change in BDI-II score, with concurrent psychological treatment, group allocation, and baseline BDI-II score entered in the initial steps. A significant moderation effect indicated that those with more severe depressive symptoms at baseline experienced a greater decrease in BDI-II score when allocated to the MemFlex condition, relative to the Psychoeducation condition, *β* = −1.26, *p* = .007, Δ*R*^2=^ 0.11, *F*(1, 47) = 8.11, *p* = .007.

## 3 Discussion

The primary aim of this early-phase RCT was to estimate the likely size of the effects of MemFlex on memory flexibility and depressive symptoms, relative to Psychoeducation, a low-intensity intervention option currently endorsed in the NICE guidelines for depression and routinely offered as a first-line intervention in the UK NHS. Both Psychoeducation and MemFlex produced reductions in depressive symptoms. MemFlex produced non-significant, small to moderate effect sizes, relative to Psychoeducation, on our cognitive target of AMT-AI performance, our co-primary clinical outcome of diagnostic status, and our secondary clinical outcome of depression-free days. On average, those completing MemFlex achieved an additional two weeks depression-free over a three month follow-up period. There was a small effect in favour of MemFlex for our co-primary measure of self-reported depressive symptoms. Those in the MemFlex condition experienced minimally clinically significant improvement (29%) on self-reported symptoms (using criteria defined by Button et al., 2015) with 64% of participants no longer meeting criteria for a MDE at three month follow-up, had a low drop-out rate (16%), and rated MemFlex as likely to yield greater improvement in symptoms than Psychoeducation (*d* = 0.45).

As this was an early-stage trial, we aimed to estimate likely effect sizes to adequately power a larger, later-stage trial which would determine the statistical and clinical significance of treatment effects. Such a trial is clearly necessary before implications can be drawn regarding treatment efficacy. The current effect size estimates in favour of MemFlex relative to an established low-intensity intervention suggest that MemFlex may have both a larger effect on depressive outcomes, and also shift an additional risk factor for relapse (Sumner et al., 2010) – impaired retrieval of autobiographical memories. Furthermore, treatment effect sizes for MemFlex were similar in size to that consistently observed for current evidence-based, guided self-help treatments such as computerised-CBT (for meta-analysis see Richards & Richardson, 2012). These effect sizes may now be used to inform a power calculation for a fully-powered definitive RCT evaluating the efficacy of MemFlex as a low-intensity intervention option for treatment of depression.

Although MemFlex delivered larger effect sizes for both the cognitive target of memory flexibility and the clinical outcomes, these were non-significant, as was expected in this early-phase trial. In our exploratory analyses we only found a very small effect size for the indirect effect on depression through memory flexibility. A future trial should therefore include an embedded mechanism study in order to further investigate the putative processes underlying therapeutic change. Our results demonstrated little support for cognitive avoidance and rumination, and an effect size in favour of Psychoeducation for problem solving, which is perhaps unsurprising given that the workbook sessions included writing out plans to address a range of identified issues and therefore trained problem solving directly. However, our recent experimental work indicated that the accessibility of autobiographical memories plays an important role in appropriately restraining negative self-judgements (Hitchcock et al., 2017a). As overgeneralised negative self-beliefs drive depressive symptoms (Beck, 1967), it may be important to explore negative self-appraisals as a mechanism underlying the effect of MemFlex. Our preliminary moderation analysis also suggested that MemFlex may have larger treatment effects for those with more severe symptoms, which will need to be further evaluated in the fully-powered RCT. Such an RCT should ensure that clinicians delivering the intervention are not immediately supervised by those who created the intervention, to address any potential for allegiance and transportability effects.

The primary aim of low-intensity interventions such as Psychoeducation or Memflex is to improve the accessibility and cost-effectiveness of psychological treatment. The fact that across all participants 55% achieved minimally clinically significant improvement (Button et al., 2015) and 50% no longer met criteria for a current MDE suggests a role for these interventions as part of broader systems of care provision. We did not have a non-intervention comparison group to establish baseline levels of spontaneous remission, which would have aided clarification of non-specific treatment effects. However, meta-analysis of spontaneous recovery in waitlist control conditions suggests a 15.7% mean decrease in BDI scores from pre-to post-assessments; that is, less than that observed in our results. Our examined interventions are therefore both likely to improve upon natural recovery (as indexed by waitlist recovery trajectories), although direct comparison to a waitlist-control condition is necessary.

Although low-intensity treatments such as MemFlex are unlikely to resolve complex and comorbid presentations, they could either be utilised as part of a stepped-care model (Bower & Gilbody, 2005) or potentially combined with more intensive psychological interventions to tailor treatment toward the specific risk factors experienced by the individual (in this case, maladaptive autobiographical processing styles). Indeed, the primary rationale for development of MemFlex was to improve therapeutic intervention into one of the underlying cognitive factors which predicts the recurrence of depression (i.e., flexibility of autobiographical memory retrieval), but does not appear to be commonly shifted by existing treatment options. The primarily workbook-based format of such interventions lends well to delivery as an adjunct to a more complex intervention, or for those on a waiting list for intervention. Indeed, completion of MemFlex while waiting for CBT may help build the skills necessary for later cognitive restructuring by improving the ability to access specific positive information which can be used as evidence to counter a negative belief.

In summary, we have provided early-phase trial data providing preliminary support for the potential of an autobiographical memory-based intervention, MemFlex, which has been translated from basic science as an alternative to current low-intensity depression treatments. MemFlex yielded numerically larger, though non-significant, point effect size estimates in the small to moderate range relative to Psychoeducation, a common, first-response psychological intervention. Obtained effects sizes also suggested that MemFlex may shift an additional risk factor for depressive relapse – impoverished memory flexibility-to a similar extent. The primarily workbook-based format of the programme, and the fact that it can be delivered by individuals without extensive training in psychological therapy, may help to improve the cost-effectiveness and thereby accessibility of psychological interventions for depression. A larger-scale, fully-powered definitive trial with an embedded mechanism study is now indicated to determine potential efficacy relative to other low-intensity alternatives, and further evaluate the mechanism of change.

## Footnotes

1 Please note that baseline data for 34 of the participants contributed to a separate study comparing performance on the Alternating Instructions version of the Autobiographical Memory Test between healthy and depressed individuals (see preprint; Hitchcock et al., 2018). These participants were randomised between MemFlex and Psychoeducation, and no significant differences were observed between those who had and had not contributed data to the additional study for memory performance, depressive symptoms, and demographics, *F*s < 1.

2 If an analysis of covariance on a continuous outcome produced residuals that did not achieve normality, then log, square root and reciprocal transformations were used and normality of the residuals re-assessed using both the Kolmogorov-Smirnov and Shapiro-Wilk tests. Heterogeneity of group variance was assessed using Levene's test and, if found, robust Huber-White standard errors were used (Huber, 1967). For our analysis of primary outcomes, the use of transformations failed to induce normality in the residuals, and as such, group differences were analysed using Quade's rank test of analysis of covariance (1967).

3 Two individuals scored ≥13 at their eligibility screening, and then on the day of the baseline assessment, scored 10 and 12 respectively on the BDI-II. Importantly, these individuals still met DSM criteria for current Major Depressive Episode. Analyses were re-run without these individuals, and results remained the same.

4 We did not covary pre-intervention BDI-II scores in our primary analysis, in accordance with our published protocol paper which specified that only measures with significant between-group differences at baseline would be used as covariates. However, due to the moderate but non-significant baseline difference in BDI-II scores we re-ran this analysis to co-vary these pre-intervention scores. The results were as follows: *t* = 0.14, *p* = .89, *d* = 0.06 [-0.47, 0.59].
