# Supplementary material for: A randomised controlled trial of memory flexibility training (MemFlex) to enhance memory flexibility and reduce depressive symptomatology in individuals with major depressive disorder
Source: Behav Res Ther. 2018 Nov;110:22–30. doi: 10.1016/j.brat.2018.08.008 (PMC6173798; doi:10.1016/j.brat.2018.08.008)
Supplement: Multimedia component 1 [file mmc1.doc]

Supplementary Table 1. *Correlations between cognitive target, clinical outcomes, and process measures at baseline.*

|  | 1. | 2. | 3. | 4. | 5. | 6. | 7. |
| --- | --- | --- | --- | --- | --- | --- | --- |
| 1. Beck Depression Inventory II | - | .011 | -.049 | -.038 | .404^**^ | .360^*^ | -.082 |
| 2. Total proportion correct for AMT-AI |  | - | .311^*^ | .433^**^ | .129 | -.116 | .464^**^ |
| 3. MEPS - Means |  |  | - | .860^**^ | -.056 | -.220 | .275^*^ |
| 4. MEPS - Effectiveness |  |  |  | - | -.117 | -.167 | .276^*^ |
| 5. Rumination |  |  |  |  | - | .081 | -.116 |
| 6. Cognitive Avoidance |  |  |  |  |  | - | -.287^*^ |
| 7. Verbal Fluency |  |  |  |  |  |  | - |

* *p* ≤ .05 ** *p* ≤ .001

AMT-AI = Alternating Instructions Autobiographical Memory Test (Dritschel et al., 2014)

MEPS = Means Ends Problem Solving Task (Lyubomirsky & Nolen-Hoeksema, 1995)
